# Preclinical Prediction of Resistance Mutations and Proposal of Sequential Treatment Strategies for ALK-positive Lung Cancer Using Next-generation ALK Inhibitors

**DOI:** 10.1007/s11095-025-03916-1

**Published:** 2025-09-24

**Authors:** Yuki Takei, Hirotaka Kuroiwa, Chisaki Arai, Yuta Doi, Kentaro Semba

**Affiliations:** 1 Department of Life Science and Medical Bioscience, Graduate School of Advanced Science and Engineering, Waseda University, 2‑2 Wakamatsu‑cho, Shinjuku‑ku, Tokyo, 162‑0056 Japan; 2 Translational Research Center, Fukushima Medical University, Hikarigaoka, Fukushima 960‑1295 Japan

**Keywords:** ALK, Lung cancer, Tyrosine kinase

## Abstract

**Background:**

Anaplastic lymphoma kinase (ALK) gene rearrangements occur in approximately 5% of non-small cell lung cancers (NSCLCs). Although ALK tyrosine kinase inhibitors provide substantial clinical benefits, acquired resistance-conferring mutations frequently emerge, leading to disease progression. Preclinical prediction of these mutations might help guide the development of more effective sequential treatment strategies prior to clinical application.

**Objective:**

To predict the emergence of resistance mutations to the investigational ALK inhibitors zotizalkib (TPX-0131), gilteritinib (ASP2215), and neladalkib (NVL-655) following resistance to first-line alectinib and assess the potential of these drugs as second-line therapies.

**Methods:**

A polymerase chain reaction (PCR)-based mutagenesis system was used to introduce random mutations into ALK cDNA harboring representative alectinib-resistant mutations. Mutant libraries were expressed in Ba/F3 cells, which were exposed to each inhibitor. Drug-resistant clones were isolated, sequenced, and evaluated for drug sensitivity using viability assays and immunoblotting.

**Results:**

Several resistance mutations against zotizalkib, gilteritinib, and neladalkib were identified. Sequential use of these agents effectively suppressed all predicted resistance patterns with G1202R or I1171N.

**Conclusions:**

This PCR-based platform provides a valuable approach for anticipating resistance mutations and guiding the design of optimized sequential therapies. Zotizalkib, gilteritinib, and neladalkib might represent promising alternatives to lorlatinib as second-line treatments for ALK-positive NSCLC.

**Key Points:**

• A PCR-based mutation prediction system was successfully applied to fourth-generation ALK inhibitors.

• Neladalkib showed efficacy against G1202R-positive relapses with minimal evidence of secondary resistance mutations.

• Sequential combinations of gilteritinib with either neladalkib or ensartinib may sustain efficacy and delay resistance in I1171N-positive relapses.

**Supplementary Information:**

The online version contains supplementary material available at 10.1007/s11095-025-03916-1.

## Introduction

The anaplastic lymphoma kinase (ALK) gene has been recognized as a tumor-agnostic oncogene, and the term “ALKoma” has been proposed to reflect the oncogenic activation of ALK across several malignancies. ALK activation occurs through multiple mechanisms, including point mutations, gene amplification, and chromosomal rearrangement [1]. Lung cancer remains the leading cause of cancer-related mortality globally, accounting for approximately 1.8 million deaths annually, and ALK rearrangements have been identified in approximately 5% of non-small cell lung cancers (NSCLCs) [2, 3]. In most cases of ALK-rearranged NSCLC, an inversion on chromosome 2 leads to the fusion of the ALK gene with the echinoderm microtubule-associated protein-like 4 (EML4) gene [4]. The resulting EML4–ALK fusion protein remains constitutively active through the oligomerization (coiled-coil) domain of EML4, driving downstream signaling pathways that promote uncontrolled cell proliferation and tumor progression [5–7].

Multiple ALK tyrosine kinase inhibitors (ALK-TKIs) have been developed for ALK-positive NSCLC. Crizotinib, the first ALK-TKI approved for clinical use, achieved better progression-free survival than standard chemotherapy in phase III trials [8]. Subsequently, alectinib, a second-generation ALK-TKI, exhibited even greater efficacy, and it has become the preferred first-line treatment for ALK-positive NSCLC [9]. However, resistance commonly develops within a few years, often because of secondary point mutations in the ALK kinase domain such as G1202R or I1171N [10].

To overcome resistance mutations, the third-generation ALK-TKI lorlatinib was developed, and it is currently recommended as a second-line treatment following alectinib failure [11, 12]. Nevertheless, compound mutations, such as G1202R + L1196M, can emerge after lorlatinib treatment, leading to further resistance and disease progression [13, 14]. In addition, lorlatinib has been linked to frequent and severe adverse effects, including hypercholesterolemia, hypertriglyceridemia, peripheral neuropathy, and cognitive impairment. These toxicities are generally more pronounced than those observed with other ALK-TKIs, thereby compromising patients’ quality of life [15]. Furthermore, some patients exhibit primary resistance to lorlatinib, underscoring the need for alternative second-line strategies. In this context, anticipating resistance mutations prior to treatment represents a promising approach to guiding the selection of effective sequential treatments.

Several groups developed resistance prediction systems using N-ethyl-N-nitrosourea (ENU)-based mutagenesis screens [14, 16]. However, ENU induces mutations through chemical modification of DNA bases, leading to a biased mutation spectrum and potentially incomplete coverage of relevant variants [17]. Furthermore, because ENU introduces genome-wide mutations, it is difficult to assess the effects of ALK mutations in isolation.

To address these limitations, we previously established a resistance prediction platform using error-prone polymerase chain reaction (PCR), which specifically introduces mutations into the ALK kinase domain. Using this system, we predicted resistance mutations that arise when repotrectinib or ensartinib is used as a second-line treatment against alectinib-resistant variants such as G1202R- and I1171N-mutated cancer [18]. However, the use of repotrectinib or ensartinib led to the emergence of mutations that could not be treated with existing drugs. Therefore, it is necessary to explore compounds, including fourth-generation ALK inhibitors, capable of preventing the emergence of untreatable resistance mutations.

In this study, we applied an error-prone PCR-based mutagenesis approach to three compounds, including the fourth-generation ALK inhibitors zotizalkib (TPX-0131) and neladalkib (NVL-655), as well as gilteritinib (ASP2215), which was originally developed for acute myeloid leukemia and is currently being evaluated in clinical trials for ALK-positive NSCLC. Our aim was to predict resistance mutations that can emerge when these compounds are used following alectinib failure. Furthermore, we assessed whether other ALK-TKIs could overcome resistance mutations that were predicted to arise after second-line treatment with zotizalkib, gilteritinib, or neladalkib and evaluated their potential utility as alternative second-line therapeutic options to lorlatinib.

## Materials and Methods

### Cell Lines and Culture Condition

Ba/F3 cells, which are murine bone marrow-derived pro-B cells, were cultured in RPMI-1640 medium (FUJIFILM Wako, Osaka, Japan) supplemented with 10% fetal bovine serum (FBS; Nichirei Biosciences, Tokyo, Japan), 100 U/mL penicillin (Meiji Seika Pharma, Tokyo, Japan), 100 µg/mL streptomycin (Meiji Seika Pharma), and 10 ng/mL murine interleukin 3 (IL-3; PeproTech, Cranbury, NJ, USA) and incubated at 37℃ in a 5% CO_2_ atmosphere. Platinum-E retroviral packaging cells (Plat-E cells) were cultured in Dulbecco’s Modified Eagle Medium (low glucose; FUJIFILM Wako) supplemented with 10% FBS, 100 U/mL penicillin, and 100 µg/mL streptomycin and incubated at 37℃ and 5% CO_2_.

### Reagents

Alectinib (CH5424802), crizotinib (PF-02341066), lorlatinib (PF-06463922), neladalkib (NVL-655), and brigatinib (AP26113) were purchased from Selleck Chemicals (Houston, TX). Zotizalkib (TPX-0131) and gilteritinib (ASP2215) were purchased from MedChemExpress (Monmouth Junction, NJ, USA). All inhibitors were dissolved in dimethyl sulfoxide (DMSO) and stored at − 80℃ after aliquoting.

### Construction of Complementary DNA (CDNA) Mutant Libraries Using Error‑prone PCR

We previously generated cDNA mutant libraries possessing G1202R or I1171N and a random mutation [18]. These constructs were generated by cloning the cDNA sequence of EML4–ALK variant 1 (GenBank: AB274722.1), which is the most common variant, being observed in approximately 45% of ALK-positive NSCLCs [19], into the pMXs-GW-IRES-Puro vector. The inserted sequence harbored either the G1202R or I1171N mutation. Error-prone PCR targeting the ALK kinase domain was subsequently performed to introduce random mutations. We used this library for this study. Detailed methods were described previously [18].

### Establishment of Ba/F3 Cells Expressing cDNA Mutant Libraries

For retrovirus production, Plat-E cells (2 × 10^6^ cells) were seeded into a 100-mm cell culture dish. After overnight culture, 10 μg of the established cDNA mutant Library and 30 μg of polyethylenimine were added to the culture. After incubation for 8 h, the medium was replaced with RPMI-1640. After another 24 h of incubation, the cell culture containing retrovirus was harvested, and cell debris was removed via centrifugation for 15 min at 3500 rpm. Cultured Ba/F3 cells (1 mL; 5 × 10^6^ cells/well, three wells in total) were seeded into 12-well plates and mixed with 1 mL of the harvested retrovirus supplemented with 8 μg/mL polybrene for spinfection. The Ba/F3 cells were centrifuged in the 12-well plates for 1 h at 32℃ (900 × *g*). Thereafter, all cultures were transferred to 25-cm^2^ cell culture flasks, and 9 mL of the mixture of RPMI-1640 medium and the harvested virus supplemented with 8 μg/mL polybrene were added. After overnight incubation at 37℃ and 5% CO_2_, the virus was removed via washing and centrifugation, and the cells were transferred to 30 mL of RPMI-1640 medium supplemented with 0.5 ng/mL IL-3 in a 75-cm^2^ culture flask. After 24 h of incubation, the culture medium was changed to 60 mL of RPMI-1640 supplemented with 0.05 ng/mL IL-3 and 1 μg/mL puromycin (FUJIFILM Wako) in a 75-cm^2^ culture flask. Puromycin was applied to Ba/F3 cells for 48 h before establishing the cDNA mutant library of Ba/F3 cells. To assess viral infection efficiency, the infected cells were transferred to six-well plates (2 × 10^5^ cells/mL in 2 mL of culture), and puromycin (final concentration 1 μg/mL) was then added to each well individually. Living cells were monitored using a hemocytometer by staining dead cells with 0.5% trypan blue over 40 h after the addition of puromycin when non-infected cells were completely dead. The infection efficiency was calculated by drawing the growth curve of the cells and predicting the percentage of infected cells against the total number of living cells before the addition of puromycin.

### Identification of Resistance Mutations Against Zotizalkib, Gilteritinib, or Neladalkib

Ba/F3 cells (1000 cells) expressing EML4–ALK and carrying G1202R and a random mutation were seeded into 96-well plates and cultured with zotizalkib (100 nM) for 2 weeks. Ba/F3 cells (1000 cells) expressing EML4–ALK and carrying I1171N and a random mutation were seeded into 96-well plates and cultured with gilteritinib (100 nM) for 2 weeks. Ba/F3 cells (20,000 cells) expressing EML4-ALK and carrying G1202R and a random mutation were seeded into 96-well plates and cultured with neladalkib (50 nM) for 2 weeks. Ba/F3 cells (1000 cells) expressing EML4-ALK and carrying I1171N and a random mutation were seeded into 96-well plates and cultured with neladalkib (500 nM) for 2 weeks. Drug-resistant clones were expanded, and the regions encoding the ALK kinase domain were then amplified from the resistant mutants using KOD FX Neo polymerase (TOYOBO, Osaka, Japan). Mutations were detected by Sanger sequencing. For the cell viability assay, Ba/F3 cells expressing EML4–ALK and harboring the predicted resistance mutation were established as described in Section "Establishment of Mutated EML4–ALK-expressing Ba/F3 Cells". Base substitutions were replicated according to the results of Sanger sequencing. For retrovirus infection, the initial culture size was reduced to 2 mL, and instead of spinfection, Ba/F3 cells (4 × 10^5^ cells) were cultured for 24 h in a mixture of 1 mL of RPMI-1640 medium and 1 mL of retrovirus-containing medium supplemented with 8 µg/mL polybrene.

### Establishment of Mutated EML4–ALK-expressing Ba/F3 Cells

The predicted resistant mutations were generated in the pMXs-GW-IRES-Puro vector containing the cDNA of EML4–ALK variant 1 plus the G1202R or I1171N mutation using the PrimeSTAR® Mutagenesis Basal Kit (TaKaRa Bio Inc., Shiga, Japan). Subsequently, the product was transformed into competent DH5α *Escherichia coli* cells via 1 min of heat shock at 42℃. The *E. coli* cells were incubated in super optimal broth with catabolite repression medium (2% tryptone, 0.5% yeast extract, 10 mM NaCl, 2.5 mM KCl, 20 mM MgSO_4_, and 20 mM glucose) for 1 h with intense shaking and then seeded on lysogeny broth (LB) agar plates supplemented with 100 mg/L ampicillin. After overnight incubation, colonies were individually collected and replicated onto other LB agar plates. Colony PCR was performed against the collected colonies. The regions encoding the ALK kinase domain were amplified from the resistant mutants using KAPA Taq Extra DNA polymerase (KAPA Biosystems, Potters Bar, UK), and individual base substitutions were confirmed via Sanger sequencing. Plasmid DNA was prepared using the FastGene™ Plasmid Mini Kit (NIPPON Genetics Co., Ltd, Tokyo, Japan).

### Cell Viability Assay

Ba/F3 cells were seeded into 96-well plates (2000 or 4000 cells/well, 90 μL), and 10 μL of serially diluted inhibitors were added to the culture. Three wells were prepared to evaluate cell viability at each drug concentration. Three additional wells were prepared for negative controls by adding 90 μL of the medium and 10 μL of DMSO. After a 72-h incubation period at 37℃, each culture was mixed with 10 μL of Cell Counting Kit-8 (CCK-8) reagent (DOJINDO, Kumamoto, Japan). After 2 h of incubation, the absorbance of the mixture at 450 nm was measured using the Synergy H1 multimode reader (BioTek Instruments, Winooski, VT, USA). The measured score in each well was subtracted from the average score in the negative controls. The score for the 0 nM drug condition was defined as a relative cell viability of 1.0, and the relative cell viability in each well was then calculated. The data were analyzed for drawing graphs, and the half-maximal inhibitory concentration (IC_50_) was then calculated from the data using GraphPad Prism version 6 (GraphPad software, Boston, MA, USA).

### Antibodies and Immunoblotting

Ba/F3 cells (1 × 10^6^ cells) were seeded into 12-well plates and treated with different concentrations of inhibitors for 3 h. PBS was used to wash the cells, which were then suspended in TNE buffer (10 mM Tris–HCl [pH 7.4], 1 mM EDTA, 150 mM NaCl, and 1% NP-40). The total protein concentration was measured using the Pierce™ BCA Protein Assay Kit (Thermo Fisher Scientific, Waltham, MA, USA). The suspension was then treated with 2 × sample buffer containing 100 mM Tris–HCl (pH 6.8), 4% sodium dodecyl sulfate (SDS), 20% glycerol, 10% 2-mercaptoethanol, and 0.01% bromophenol blue. The samples were thoroughly sonicated and boiled at 95℃ for 5 min. Furthermore, 5 μg of the total protein of the samples were electrophoresed in 7.5% SDS–polyacrylamide gels. The protein was transferred from the gels to Immobilon PVDF membranes (Merck Millipore Ltd., Burlington, MA, USA). The membranes were immersed in appropriate blocking buffer (Tris-buffered saline with 0.05% Tween 20 [TBST] supplemented with 5% w/v bovine serum albumin or 5% w/v nonfat dry milk) for 1 h at room temperature. Subsequently, the membranes were incubated overnight at 4℃ with gentle agitation in primary antibody dilution buffer (phosphorylated ALK [Y1604; Cell Signaling Technologies, Danvers, MA, USA; #3341, 1:1000], ALK [Cell Signaling Technologies; #3791, 1:2000], and α-tubulin [FUJIFILM Wako; #013-25033, 1:4000]). After washing with TBST, the membranes were incubated for 1 h at room temperature with gentle agitation in blocking buffer supplemented with appropriate horseradish peroxidase (HRP)-linked secondary antibodies (anti-rabbit immunoglobulin G [IgG], HRP-linked antibody [Cell Signaling Technologies; #7074, 1:4000], and anti-mouse IgG, HRP-linked antibody [Cell Signaling Technologies; #7076, 1:4000]). The membranes were then washed with TBST and incubated with Immobilon® Western Chemiluminescent HRP Substrate (Merck Millipore Ltd.) or ImmunoStar® LD (FUJIFILM Wako) at room temperature for 3 min. Protein was detected using the ChemiDoc XRS+System (Bio-Rad Laboratories, Hercules, CA, USA).

## Results

### Sensitivity of Alectinib‑resistant Mutants to Zotizalkib, Gilteritinib, and Neladalkib

**Fig. 1:**
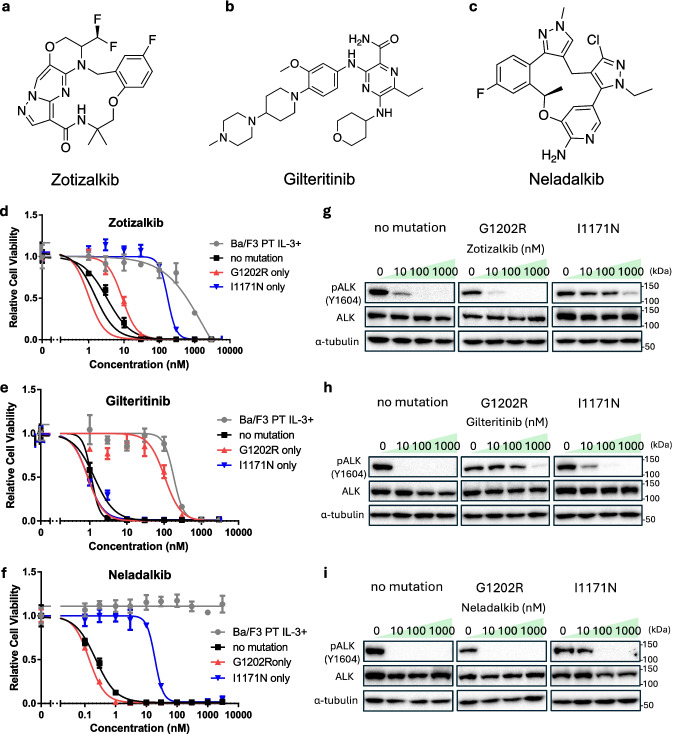
The sensitivity of alectinib-resistant mutants against zotizalkib, gilteritinib, and neladalkib. **a** Chemical structure of zotizalkib. **b** Chemical structure of gilteritinib. **c** Chemical structure of neladalkib. **d–f** Sensitivity evaluation of alectinib-resistant mutants against zotizalkib (**d**), gilteritinib (**e**), and neladalkib (**f**). Ba/F3 cells expressing EML4–ALK variant 1 (v1) plus G1202R or I1171N were exposed to each inhibitor for 72 h. Cell viability was evaluated by CCK-8, with absorbance measured at 450 nm. **g–i** Immunoblotting evaluation of the suppression of phosphorylated ALK expression in alectinib-resistant mutants by zotizalkib (**g**), gilteritinib (**h**), or neladalkib (**i**). Ba/F3 cells expressing EML4–ALK v1 and each resistance mutation were treated with different inhibitors for 3 h. Next, immunoblotting was used to detect the indicated protein in cell lysates. *CCK-8* Cell Counting Kit-8, *EML4* echinoderm microtubule-associated protein-like 4, *ALK* anaplastic lymphoma kinase.

Alectinib is firmly established as the standard first-line treatment for ALK-positive NSCLC in Japan. However, disease progression inevitably occurs in many patients because of the emergence of resistance mutations. To evaluate the potential of emerging ALK inhibitors as second-line therapies, we first assessed their efficacy against common alectinib-resistant mutations. We established Ba/F3 cells expressing EML4–ALK variant 1 harboring either the G1202R or I1171N mutation and tested their sensitivity to three next-generation ALK-TKIs: zotizalkib, gilteritinib, and neladalkib (Fig. 1a–c). CCK-8 cell viability assays revealed that the G1202R mutant was sensitive to zotizalkib and neladalkib but resistant to gilteritinib, whereas the I1171N mutant was sensitive to gilteritinib and neladalkib but resistant to zotizalkib (Fig. 1d–f, Table I), consistent with previous reports [18, 20–22]. Immunoblotting confirmed that zotizalkib and gilteritinib inhibited ALK phosphorylation in cells carrying the G1202R and I1171N mutations, respectively, whereas neladalkib inhibited ALK phosphorylation in both cell lines (Fig. 1g–i). Based on these results, we selected all three drugs for further evaluation as potential sequential treatment options following alectinib.

**Table I:** IC_50_s of ALK-TKIs in Ba/F3 Cells Carrying Alectinib-resistant Mutations

IC_50_ (nM)			
	Zotizalkib	Gilteritinib	Neladalkib
Ba/F3 PT IL-3+	1630	182.6	N.D
fold change in IC_50_	6.5	1.1	0.2
G1202R	9.0	107.1	0.1
fold change in IC_50_	(× 1.3)	(× 97)	(× 0.5)
I1171N	190.7	0.9	19.1
fold change in IC_50_	(× 29)	(× 0.8)	(× 95)

IC_50_s were calculated from the results of the cell viability assay using GraphPad Prism version 6 (Fig. 1d-f). The fold change in IC_50_ relative to the no mutation is shown below each IC_50_ value. *IC*_*50*_ half-maximal inhibitory concentration, $ALK$ anaplastic lymphoma kinase, $TKIs$ tyrosine kinase inhibitors, *N.D.* not determined

### Experimental System for Predicting Resistance Mutations Using Error‑prone PCR

Acquired point mutations in the ALK kinase domain represent the primary mechanism of resistance following ALK-TKI therapy. Therefore, predicting resistance mutations at the preclinical stage is important for developing effective therapies following alectinib treatment. To address this, we previously established a novel preclinical prediction platform using error-prone PCR [18]. In the present study, the cDNA Libraries were transfected into Plat-E cells, packaged into retroviruses, and used to infect 6× 10^7^ Ba/F3 cells. After puromycin selection, the estimated infection efficiencies were 3.44% (zotizalkib, G1202R), 2.53% (gilteritinib, I1171N), 21.4% (neladalkib, G1202R), and 10.8% (neladalkib, I1171N), as presented in Supplementary Fig. 1a. As the cDNA mutant Library was constructed from approximately 7× 10^5^ colonies and that more than 1 × 10^6^ Ba/F3 cells were infected per condition, it is likely that the full spectrum of possible point mutations was represented in the transduced cell populations.

### Prediction of Zotizalkib-Resistant Mutations and Sensitivity to Other ALK-TKIs

**Fig. 2:**
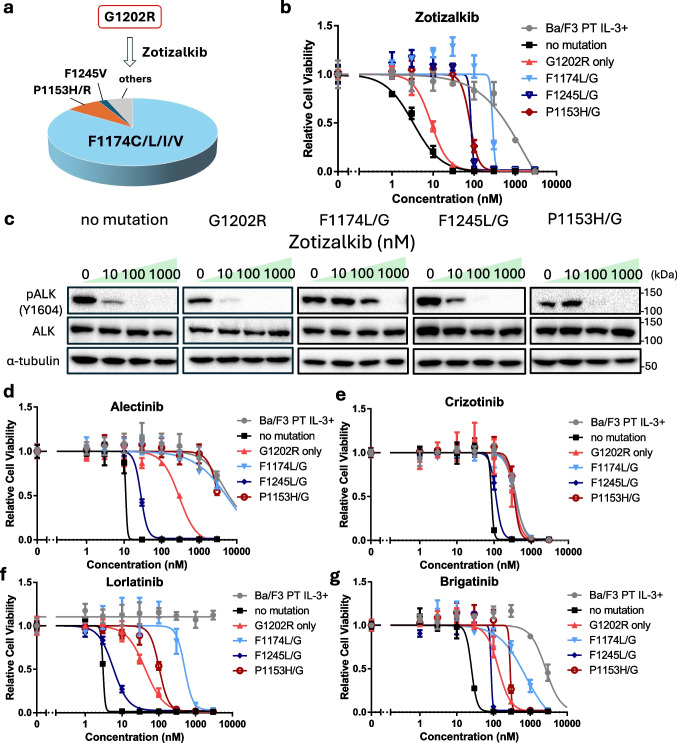
Zotizalkib-resistant mutations emerging from G1202R-positive ALK and their cross-sensitivity to other ALK-TKIs. **a** Predicted zotizalkib-resistant mutations (*n* = 54). Mutant libraries of Ba/F3 cells were exposed to zotizalkib (100 nM) for 2 weeks. **b** Sensitivity evaluation of predicted zotizalkib-resistant mutants against zotizalkib. Ba/F3 cells expressing EML4–ALK variant 1 and a zotizalkib-resistant mutation were exposed to zotizalkib for 72 h. Cell viability was evaluated using the CCK-8 assay, with absorbance measured at 450 nm. **c** Immunoblotting evaluation of the suppression of phosphorylated ALK expression in predicted zotizalkib-resistant mutants by zotizalkib. Ba/F3 cells expressing EML4–ALK variant 1 and a resistance mutation were treated with zotizalkib for 3 h. Next, immunoblotting was used to detect the indicated protein in cell lysates. **d–g** Sensitivity evaluation of predicted zotizalkib-resistant mutants against alectinib (**d**), crizotinib (**e**), lorlatinib (**f**), and brigatinib (**g**). Ba/F3 cells expressing EML4–ALK variant 1 with each resistance mutation were exposed to zotizalkib for 72 h. Cell viability was evaluated by CCK-8 and absorbance at a wavelength of 450 nm. *CCK-8* Cell Counting Kit-8, *EML4* echinoderm microtubule-associated protein-like 4, *ALK* anaplastic lymphoma kinase, *TKIs* tyrosine kinase inhibitors.

First, we predicted resistance mutations to zotizalkib. A Ba/F3 mutant library, in which each cell harbored the G1202R mutation along with a random secondary mutation, was treated with 100 nM zotizalkib for 2 weeks. Under these conditions, sensitive cells were expected to be completely eliminated, as indicated by the results of the cell viability assay for alectinib-resistant mutants treated with zotizalkib (Fig. 1d). Cells were seeded at a low density into 96-well plates to isolate individual resistant clones. After 2 weeks of drug exposure, Sanger sequencing of the surviving clones revealed several compound mutations associated with zotizalkib resistance, including G1202R + F1174L/C/I/V, G1202R + F1245V, and G1202R + P1153H (Fig. 2a). Clinically, the compound mutation G1202R + F1174L has been reported as a lorlatinib-resistant mutation [23]. A point mutation at F1245 has been identified in patients with neuroblastoma [24]. Meanwhile, the P1153H mutation has not been previously reported.

Next, we investigated whether the predicted point mutations conferred resistance to zotizalkib. Ba/F3 cells expressing EML4–ALK and each of the predicted zotizalkib-resistant mutations were established, and their drug sensitivity was evaluated. These additional point mutations conferred greater resistance to zotizalkib than G1202R alone (Fig. 2b, c). To identify potential therapeutic strategies following zotizalkib treatment, we further evaluated the sensitivity of the acquired zotizalkib-resistant mutants to various ALK-TKIs. Both the G1202R + F1174L and G1202R + P1153H mutants were resistant to alectinib (Fig. 2d), crizotinib (Fig. 2e), lorlatinib (Fig. 2f), and brigatinib (Fig. 2g), whereas the G1202R + F1245L mutant remained sensitive to all of these ALK-TKIs.

### Prediction of Gilteritinib-resistant Mutations and Sensitivity to Other ALK-TKIs

**Fig. 3:**
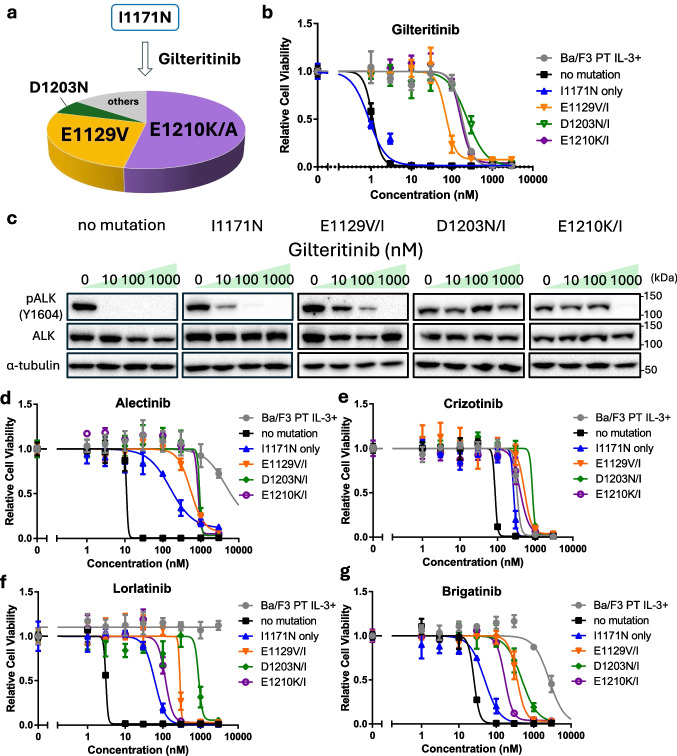
Gilteritinib-resistant mutations emerging from I1171N-positive ALK and their cross-sensitivity to other ALK-TKIs. **a** Predicted gilteritinib-resistant mutations (*n* = 49). Mutant libraries of Ba/F3 cells were exposed to gilteritinib (100 nM) for 2 weeks. **b** Sensitivity of the predicted resistant mutants to gilteritinib. Ba/F3 cells expressing EML4–ALK variant 1 and each mutation were exposed to gilteritinib for 72 h. Cell viability was evaluated by the CCK-8 assay, with absorbance measured at 450 nm. **c** Immunoblotting evaluation of the suppression of phosphorylated ALK expression in each resistant mutant by gilteritinib. Gilteritinib was given to Ba/F3 cells expressing EML4-ALK variant 1 with each resistance mutation for 3 h. Next, immunoblotting was used to detect the indicated protein in cell lysates. **d–g** Sensitivity evaluation of each resistance mutants against alectinib (**d**), crizotinib (**e**), lorlatinib (**f**), and brigatinib (**g**). Ba/F3 cells expressing EML4-ALK variant 1 with each resistance mutation were exposed to zotizalkib for 72 h. Cell viability was evaluated by the CCK-8 assay, with absorbance measured at 450 nm. *CCK-8* Cell Counting Kit-8, *EML4* echinoderm microtubule-associated protein-like 4, *ALK* anaplastic lymphoma kinase, *TKIs* tyrosine kinase inhibitors.

Next, we predicted gilteritinib-resistant mutations using the same procedure described in Section "Prediction of Zotizalkib-Resistant Mutations and Sensitivity to Other ALK-TKIs". A Ba/F3 mutant library, in which each cell harbored the I1171N mutation along with a random secondary mutation, was treated with 100 nM gilteritinib for 2 weeks. The compound mutations I1171N + E1210K/A, I1171N + E1129V, and I1171N + D1203N were identified as gilteritinib-resistant mutations (Fig. 3a). Single-point mutations at E1129, E1210, and D1203 were previously reported in patients who developed resistance to ALK-TKIs [25, 26]. Using the same validation approach described in Section "Prediction of Zotizalkib-Resistant Mutations and Sensitivity to Other ALK-TKIs", we confirmed that these additional point mutations increased resistance to gilteritinib compared with the effects of I1171N alone (Fig. 3b, c). Furthermore, these resistant mutants exhibited cross-resistance to other ALK-TKIs (Fig. 3d–g).

### Prediction of Neladalkib-resistant Mutations and Sensitivity to Other ALK-TKIs

**Fig. 4:**
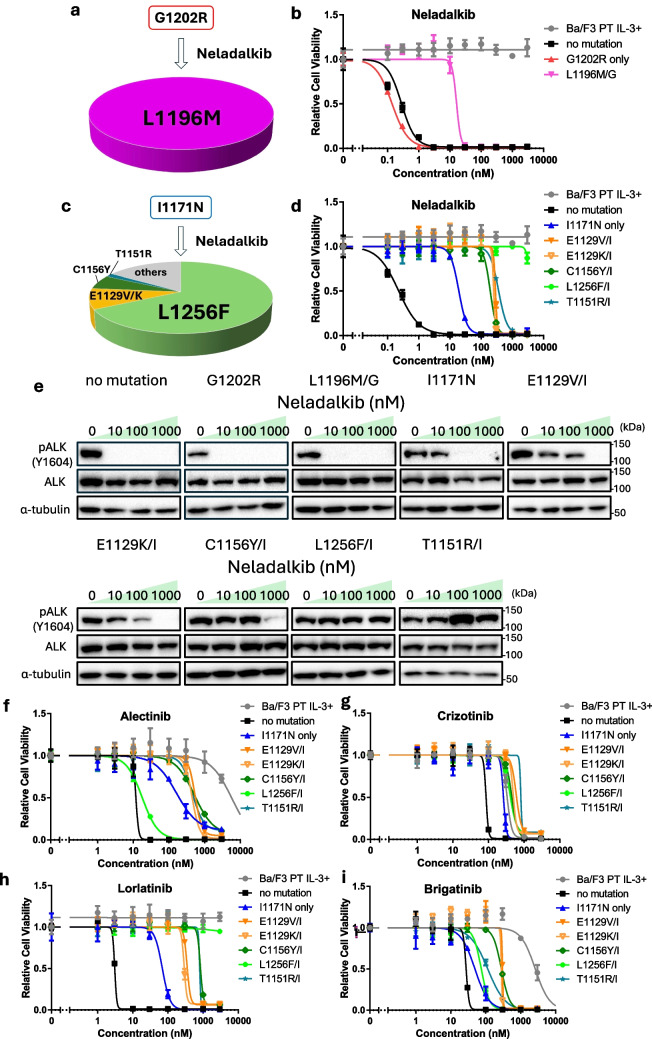
Neladalkib-resistant mutations emerging from G1202R- or I1171N-positive ALK and their cross-sensitivity to other ALK-TKIs. **a** Predicted neladalkib-resistant mutations with G1202R (*n* = 7). Mutant libraries of Ba/F3 cells were exposed to neladalkib (50 nM) for 2 weeks. **b** Sensitivity evaluation of the G1202R + L1196M compound variant against neladalkib. Ba/F3 cells expressing EML4–ALK variant 1 and different mutants were exposed to neladalkib for 72 h. Cell viability was evaluated by the CCK-8, with absorbance measured at 450 nm. **c** Predicted neladalkib-resistant mutations with I1171N (*n* = 64). Mutant libraries of Ba/F3 cells were exposed to neladalkib (500 nM) for 2 weeks. Other mutations included the triple mutation I1171N + C1156Y + D1203N, as well as I1171N + L1196M and I1171N + V1130L, both of which remained sensitive to neladalkib. **d** Sensitivity of predicted mutations to neladalkib. Ba/F3 cells expressing EML4–ALK variant 1 and different mutants were exposed to neladalkib for 72 h. Cell viability was evaluated by the CCK-8 assay, with absorbance measured at 450 nm. **e** Immunoblotting evaluation of the suppression of phosphorylated ALK expression in each resistant mutant by neladalkib. Ba/F3 cells expressing EML4–ALK variant 1 and different resistance mutations were treated with neladalkib for 3 h. Next, immunoblotting was used to detect the indicated protein in cell lysates. **f–i** Sensitivity of each resistance mutant to alectinib (**f**), crizotinib (**g**), lorlatinib (**h**), and brigatinib (**i**). Ba/F3 cells expressing EML4–ALK variant 1 and different resistance mutations were exposed to neladalkib for 72 h. Cell viability was evaluated by the CCK-8 assay, with absorbance measured at 450 nm. *CCK-8* Cell Counting Kit-8, *EML4* echinoderm microtubule-associated protein-like 4, *ALK* anaplastic lymphoma kinase, *TKIs* tyrosine kinase inhibitors.

Finally, we predicted resistance mutations to neladalkib using the procedure described in Sections "Prediction of Zotizalkib-Resistant Mutations and Sensitivity to Other ALK-TKIs" and "Prediction of Gilteritinib-resistant Mutations and Sensitivity to Other ALK-TKIs". We first screened a mutant Ba/F3 cell library harboring the G1202R mutation along with a random secondary mutation. However, even at reduced drug concentrations, extremely few cells survived. At 50 nM, only seven resistant clones were isolated, all of which harbored the G1202R + L1196M compound mutation (Fig. 4a, b). Based on the sensitivity assays, the G1202R + L1196M mutant remained responsive to neladalkib, suggesting that no additional mutations combined with G1202R conferred resistance to this compound.

Conversely, screening a Ba/F3 mutant library harboring I1171N and a random mutation at a drug concentration of 500 nM revealed several candidate resistance mutations, including I1171N + L1256F, I1171N + E1129V/K, I1171N + C1156Y, and I1171N + T1151R (Fig. 4c). The I1171N + L1256F mutation was reported as a lorlatinib-resistant mutation in an ENU-based mutagenesis screen, whereas the I1171N + C1156Y and I1171N + T1151R mutations were identified in clinical samples [10, 27, 28].

To evaluate whether these predicted point mutations conferred resistance to neladalkib, we established Ba/F3 cells expressing EML4–ALK with each mutation and assessed their drug sensitivity. These additional point mutations increased resistance to neladalkib compared with the effects of the I1171N alone (Fig. 4d, e). Furthermore, these resistant mutants exhibited cross-resistance to all tested ALK-TKIs (Fig. 4f–i).

## Discussion

The clinical introduction of multiple ALK-TKIs, including alectinib and lorlatinib, has markedly improved outcomes for patients with ALK-positive NSCLC [9]. However, disease relapse attributable to resistance mutations remains a major challenge, particularly after lorlatinib treatment [13]. As resistance profiles vary depending on the ALK-TKI used, mutation-guided sequential treatment strategies are critically needed to sustain therapeutic efficacy.

To address this issue, predicting resistance mutations that emerge after specific ALK-TKI treatments and identifying drugs effective against these variants represent a promising approach. In this study, we applied our previously established resistance mutation prediction method based on error-prone PCR [18]. Using this method, we examined the novel ALK inhibitors zotizalkib, gilteritinib, and neladalkib as second-line therapies following alectinib resistance. Zotizalkib was effective against G1202R, gilteritinib was effective against I1171N, and neladalkib was effective against both mutations (Fig. 1). Based on these results, we predicted potential compound mutations that might arise during hypothetical second-line treatment with each drug (Figs. 2, 3 and 4).

We then evaluated the sensitivity of these predicted resistance variants to each inhibitor. Although most of the variants exhibited resistance to currently approved ALK-TKIs, all variants were effectively targeted by at least one of the three investigational drugs. For compound mutations involving G1202R, neladalkib consistently exhibited potent activity, suggesting that treatment with neladalkib after G1202R arises following alectinib failure could help sustain therapeutic efficacy.

**Fig. 5:**
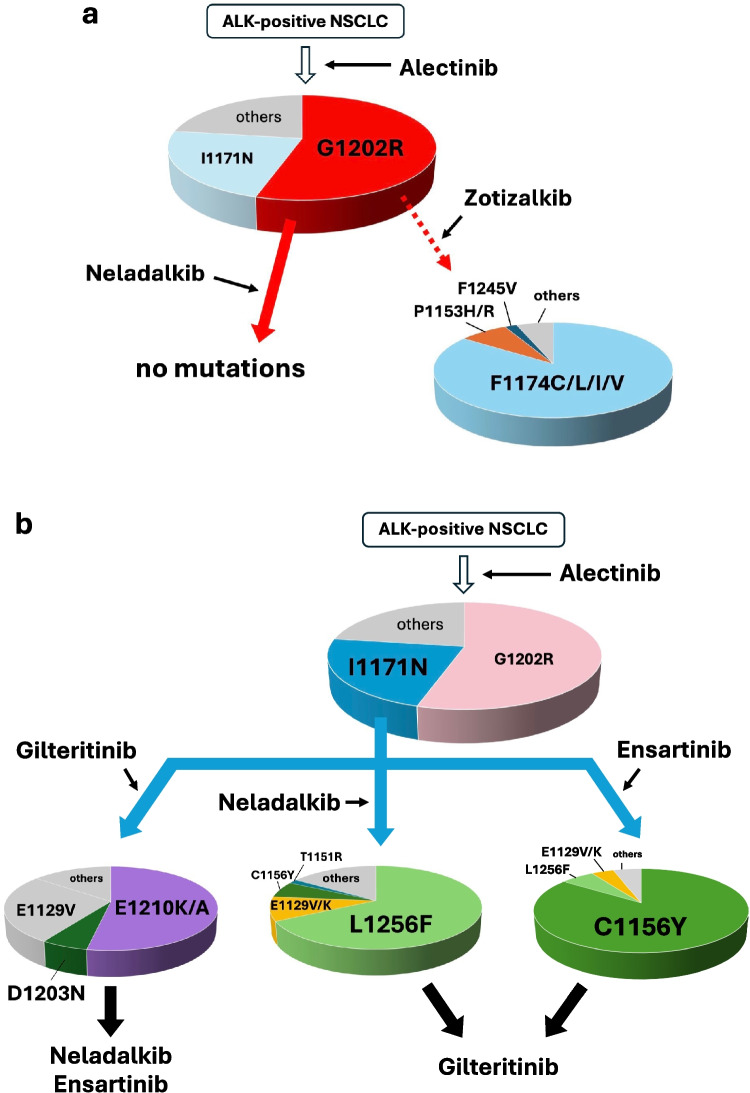
Possible therapeutic strategies after alectinib failure. **a** Neladalkib is available for G1202R-harboring ALK-positive NSCLC, and no mutations were predicted to confer resistance to neladalkib. Zotizalkib is also available for G1202R-harboring ALK-positive NSCLC; however, additional resistance mutations such as F1174C/L/I/V may emerge. Each slice size in the compound mutation pie chart reflects the proportion of mutant clones obtained. **b** Gilteritinib is a treatment option for patients with I1171N-positive ALK-rearranged NSCLC. Most relapses may occur due to an additional E1210K or D1203N mutation. In such cases, neladalkib or ensartinib may be effective. On the other hand, treatment of I1171N-mutant cells with neladalkib or ensartinib may lead to relapse due to the emergence of additional mutations such as L1256F, E1129V, or C1156Y; however, these compound mutants are considered to remain sensitive to gilteritinib. Sequential administration of gilteritinib-neladalkib or gilteritinib-ensartinib is expected to be beneficial for I1171N-positive relapses. Each slice size in the compound mutation pie chart reflects the proportion of mutant clones obtained. *ALK* anaplastic lymphoma kinase, *NSCLC* non-small cell lung cancer.

For compound mutations involving I1171N, there was no overlap in resistance profiles between gilteritinib and neladalkib, implying that sequential or combinational administration of these agents could maintain efficacy. Notably, although the I1171N + E1129V variant was predicted to confer gilteritinib resistance, its IC_50_ was 68.9 nM (Table II). Conversely, G1202R or I1171N mutants treated with lorlatinib had similar IC_50_s (approximately 90 nM), for which clinical responses have been reported [14]. Thus, this variant might have remained moderately sensitive to gilteritinib. In our previous study, we identified E1129V, C1156Y, and L1256F as secondary mutations that confer resistance to ensartinib when acquired in addition to I1171N [18]. These mutations were also associated with resistance to neladalkib; however, gilteritinib seemed to retain efficacy against them. Furthermore, compound mutations such as I1171N + E1210K and I1171N + D1203N, which confer resistance to gilteritinib, were not identified as resistant clones in our previous screening [18]. These findings suggest that these variants could retain sensitivity to ensartinib, hinting that sequential or combinational administration of ensartinib and gilteritinib could represent an effective treatment strategy for patients harboring I1171N-based resistance mutations. Taken together, our findings suggest that the use of neladalkib for G1202R-positive relapses and sequential use of gilteritinib–neladalkib or gilteritinib–ensartinib for I1171N-positive relapses could sustain treatment efficacy and delay the onset of resistance (Fig. 5).

**Table II:** IC_50_s of Several ALK-TKIs in the Presence of Various Resistance Mutations

ALK mutations	IC_50_ (nM)						
	Zotizalkib	Gilteritinib	Neladalkib	Alectinib	Crizotinib	Lorlatinib	Brigatinib
no mutation	6.5	1.1	0.2	11.1	85.2	3.0	25.2
G1202R+							
G1202R only	9.0	107.1	0.1	299.5	315.8	59.0	156.3
F1174L	277.5	-	-	> 3000	350.8	466.4	683.3
fold change in IC_50_	(× 30)	-	-	(> × 10)	(× 1.1)	(× 7.9)	(× 4.3)
F1245L	81.0	-	-	27.9	112.6	6.8	85.8
fold change in IC_50_	(× 9.0)	-	-	(× 0.09)	(× 0.3)	(× 0.1)	(× 0.5)
P1153H	77.2	-	-	> 3000	345.9	102.9	283.0
fold change in IC_50_	(× 8.5)	-	-	(> × 10)	(× 1.0)	(× 1.7)	(× 1.8)
I1171N+							
I1171N only	190.7	0.9	19.1	145.1	243.3	69.4	68.7
E1210K	-	165.4	-	849.7	402.1	116.5	167.7
fold change in IC_50_	-	(× 183)	-	(× 5.8)	(× 1.6)	(× 1.6)	(× 2.4)
D1203N	-	261.9	-	931.1	842.7	894.9	478.8
fold change in IC_50_	-	(× 291)	-	(× 6.4)	(× 3.4)	(× 12)	(× 6.9)
E1129V	-	68.9	286.0	540.1	597.9	350.6	301.5
fold change in IC_50_	-	(× 76)	(× 14)	(× 3.7)	(× 2.4)	(× 5.0)	(× 4.3)
E1129K	-	-	251.4	444.5	493.4	287.9	278.3
fold change in IC_50_	-	-	(× 13)	(× 3.0)	(× 2.0)	(× 4.1)	(× 4.0)
C1156Y	-	-	194.5	519.5	451.1	833.3	279.0
fold change in IC_50_	-	-	(× 10)	(× 3.5)	(× 1.8)	(× 12)	(× 4.0)
L1256F	-	-	> 3000	17.8	401.2	> 3000	74.0
fold change in IC_50_	-	-	(> × 157)	(× 0.1)	(× 1.6)	(> × 43)	(× 1.0)
T1151R	-	-	358.7	452.2	803.2	800.7	114.8
fold change in IC_50_	-	-	(× 18)	(× 3.1)	(× 3.3)	(× 11)	(× 1.6)

The IC_50_ was calculated according to the results of viability assays using Ba/F3 cells in this study. The fold change in IC_50_ relative to the corresponding single mutation—G1202R for G1202R-containing double mutants and I1171N for I1171N-containing double mutants—is shown below each IC_50_ value. *IC*_*50*_ half-maximal inhibitory concentration, $ALK$ anaplastic lymphoma kinase, *TKIs* tyrosine kinase inhibitors, *N.D.* not determined

## Conclusion

Using our error-prone PCR-based system, we identified potential mutations conferring resistance to zotizalkib, gilteritinib, or neladalkib. Based on these findings, we propose that these investigational drugs could represent alternative second-line treatment options to lorlatinib following alectinib failure. In clinical settings, resistance mutations are often heterogeneous, and they might not be fully captured by current diagnostic tests. Consequently, physicians might need to empirically select subsequent therapies without definitive molecular guidance. Even when molecular testing is not feasible, selecting drugs with predicted activity against plausible resistance mutations provides a rational basis for treatment decisions. When resistance mutations can be predicted, incorporating this information could enhance the precision and clinical efficacy of sequential ALK-TKI strategies. With the continued development of ALK-TKIs, we believe that individualized clinical strategies—guided by predicted resistance mutation profiles when available—could help optimize therapeutic outcomes while preserving patients’ quality of life.

## Supplementary Information

Below is the link to the electronic supplementary material.

**Sup Fig. 1** Calculation of infection efficiency **a** Cell growth after puromycin addition to calculate infection efficacy (zotizalkib, gilteritinib). The cell concentration at 0 h was 2 × 105 cells/mL. Living cells were counted at 42, 66, and 93 h after puromycin addition, and regression lines were drawn. The confidence of determination was 0.976 for zotizalkib (G1202R) and 0.997 for gilteritinib (I1171N), and the predicted initial infected cell concentration was 0.688 × 105 cells/mL for zotizalkib (G1202R) and 0.505 × 105 cells/mL for gilteritinib (I1171N). Consequently, the infection efficiency was calculated as 3.44% for zotizalkib (G1202R) and 2.53% for gilteritinib (I1171N). **b** Cell growth observation after puromycin addition to calculate infection efficacy (neladalkib). The cell concentration at 0 h was 2 × 105 cells/mL. Living cells were counted at 54, 68, and 80 h after puromycin addition, and regression lines were drawn. The confidence of determination was 0.937 for G1202R and 0.957 for I1171N, and the predicted initial infected cell concentration was 4.28 × 105 cells/mL for G1202R and 2.16 × 105 cells/mL for I1171N. Consequently, the infection efficiency was calculated as 21.4% for G1202R and 10.8% for I1171N. (PDF 76 KB)

**Sup Fig. 2** The F1245L secondary mutation restores sensitivity to ALK-TKIs in G1202R-positive ALK a–d Immunoblotting evaluation of the suppression of phosphorylated ALK expression in the presence of no mutation, G1202R alone, and G1202R + F1245L by alectinib (**a**), crizotinib (**b**), lorlatinib (**c**), or brigatinib (**d**). Ba/F3 cells expressing EML4–ALK variant 1 and different resistance mutation were treated with each inhibitor for 3 h. Next, immunoblotting was used to detect the indicated protein in cell lysates. EML4 echinoderm microtubule-associated protein-like 4, ALK anaplastic lymphoma kinase. (PDF 13550 KB)

## Data Availability

The data and material in this study are available from the corresponding author upon reasonable request.
