# Supplementary figures and images for: Preclinical Prediction of Resistance Mutations and Proposal of Sequential Treatment Strategies for ALK-positive Lung Cancer Using Next-generation ALK Inhibitors
Source: Pharm Res. 2025 Sep 24;42(9):1497–509. doi: 10.1007/s11095-025-03916-1 (PMC12508005; doi:10.1007/s11095-025-03916-1)

**a**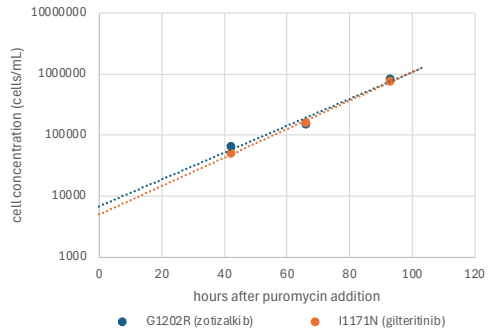**b**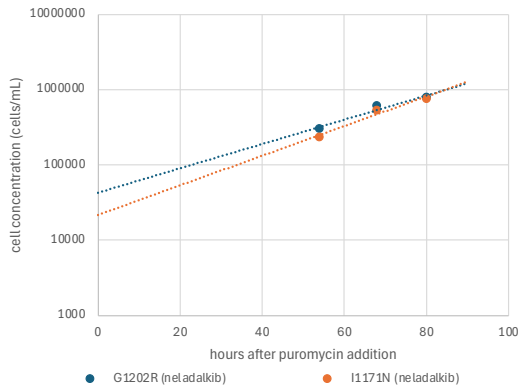

Supplement: Supplementary file 1 — Sup Fig. 1 Calculation of infection efficiency a Cell growth after puromycin addition to calculate infection efficacy (zotizalkib, gilteritinib). The cell concentration at 0 h was 2 × 10^5^ cells/mL. Living cells were counted at 42, 66, and 93 h after puromycin addition, and regression lines were drawn. The confidence of determination was 0.976 for zotizalkib (G1202R) and 0.997 for gilteritinib (I1171N), and the predicted initial infected cell concentration was 0.688 × 10^5^ cells/mL for zotizalkib (G1202R) and 0.505 × 10^5^ cells/mL for gilteritinib (I1171N). Consequently, the infection efficiency was calculated as 3.44% for zotizalkib (G1202R) and 2.53% for gilteritinib (I1171N). b Cell growth observation after puromycin addition to calculate infection efficacy (neladalkib). The cell concentration at 0 h was 2 × 10^5^ cells/mL. Living cells were counted at 54, 68, and 80 h after puromycin addition, and regression lines were drawn. The confidence of determination was 0.937 for G1202R and 0.957 for I1171N, and the predicted initial infected cell concentration was 4.28 × 10^5^ cells/mL for G1202R and 2.16 × 10^5^ cells/mL for I1171N. Consequently, the infection efficiency was calculated as 21.4% for G1202R and 10.8% for I1171N. (PDF 76 KB) [file 11095_2025_3916_MOESM1_ESM.pdf]

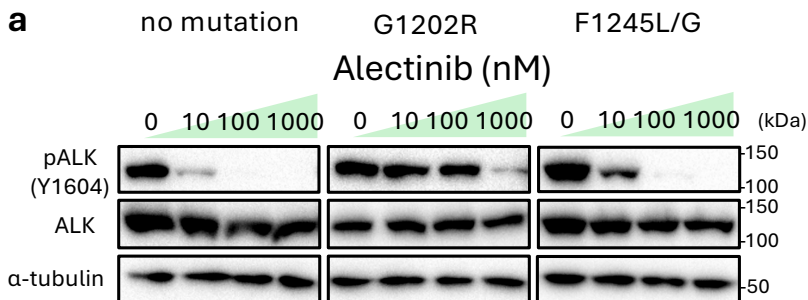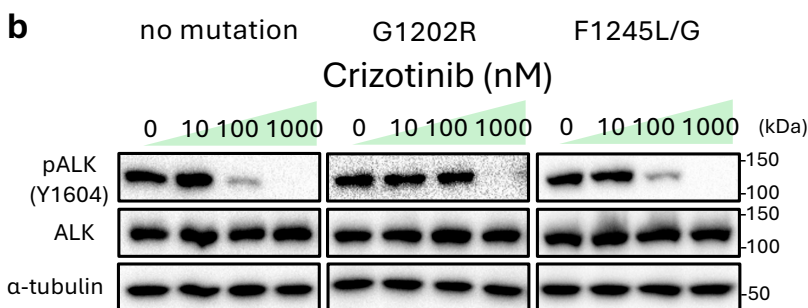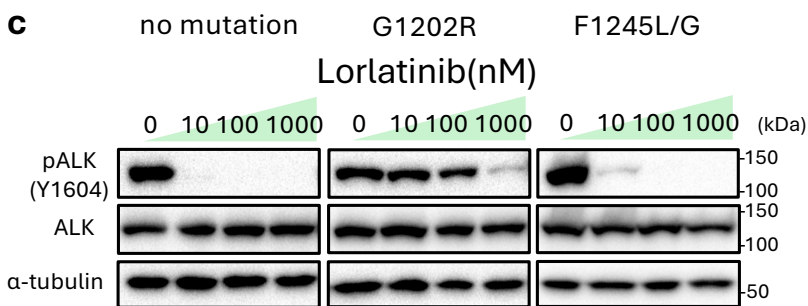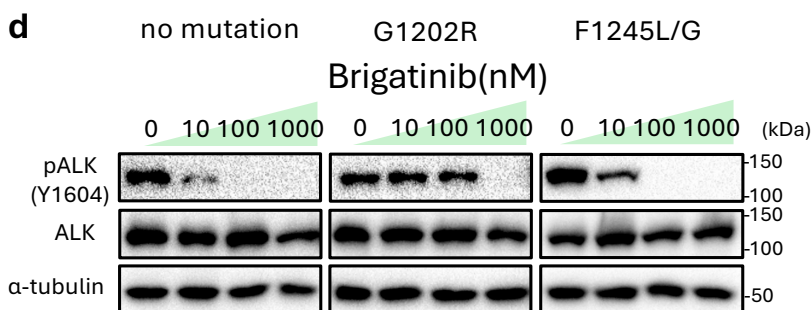

Supplement: Supplementary file 2 — Sup Fig. 2 The F1245L secondary mutation restores sensitivity to ALK-TKIs in G1202R-positive ALK a–d Immunoblotting evaluation of the suppression of phosphorylated ALK expression in the presence of no mutation, G1202R alone, and G1202R + F1245L by alectinib (a), crizotinib (b), lorlatinib (c), or brigatinib (d). Ba/F3 cells expressing EML4–ALK variant 1 and different resistance mutation were treated with each inhibitor for 3 h. Next, immunoblotting was used to detect the indicated protein in cell lysates. EML4 echinoderm microtubule-associated protein-like 4, ALK anaplastic lymphoma kinase. (PDF 13550 KB) [file 11095_2025_3916_MOESM2_ESM.pdf]
